# Impact of Venlafaxine on Platelet Count and Activity—Case Report and Narrative Review

**DOI:** 10.3390/medicina58050626

**Published:** 2022-04-30

**Authors:** Joanna Smolarczyk-Kosowska, Michał Kosowski, Łukasz Kunert, Karolina Filipczyk, Marcin Wojciechowski, Magdalena Piegza, Piotr Gorczyca, Bogusław Okopień, Robert Pudlo

**Affiliations:** 1 Department of Psychiatry, Faculty of Medical Sciences in Zabrze, Medical University of Silesia, 40-055 Katowice, Poland; lkunert@sum.edu.pl (Ł.K.); k.filipczyk@o2.pl (K.F.); marcin.woj11@gmail.com (M.W.); mpiegza@sum.edu.pl (M.P.); pgorczyca@sum.edu.pl (P.G.); rpudlo@sum.edu.pl (R.P.); 2 Department of Internal Medicine and Clinical Pharmacology, Medical University of Silesia, Medyków 18, 40-752 Katowice, Poland; mkosowski@sum.edu.pl (M.K.); bokopien@sum.edu.pl (B.O.)

**Keywords:** essential thrombocythemia, major depressive disorder, depression, SNRI, drug-induced thrombocytopenia, thrombocytopenia/chemically induced

## Abstract

Venlafaxine (VEN) is considered to be one of the most effective antidepressants. It belongs to the group of serotonin (5-HT) and noradrenaline (NA) reuptake inhibitors (SNRIs). NA and 5-HT have receptors on the surface of platelets and are involved in platelet aggregation. In this case study, we present the case of a patient treated for one of the types of myeloproliferative neoplasm (MPN), essential thrombocythemia (ET), in whom VEN was added to pharmacotherapy during the treatment of a severe episode of depression with psychotic symptoms. We observed a gradual reduction in platelet count when increasing the dose of VEN. We also present a narrative review of literature about the effect of VEN on platelet counts and activity. We conclude that, in the group of patients taking VEN, attention should be paid to the rare adverse effect of a decrease in the number of platelets.

## 1. Introduction

Myeloproliferative neoplasms (MPNs) are a group of blood cancers characterized by a benign onset but with a tendency to develop into malignant cancer in the future. According to the World Health Organization (WHO) classification, we divide MPNs into chronic myeloid leukemia (CML), chronic neutrophilic leukemia, polycythemia vera, primary myelofibrosis, essential thrombocythemia (ET), chronic eosinophilic leukemia, and unclassifiable MPNs [1]. Two of them, CML and ET, are characterized by thrombocythemia in blood tests [2]. Patients with MPNs in the daytime experience a wide range of symptoms associated with abnormal cell division in the bone marrow. The most common symptoms are fatigue, pruritus, and night sweats [3]. Fatigue is also one of the common symptoms of depression [4]. A large international study shows fatigue as one of the most common symptoms of myeloproliferative neoplasm, and proves that in the ingroup of patients with this type of proliferative disease, the risk of developing major depressive disorder (MDD) is almost 25% [5]. Another study prepared by Padrnos L. et al. also shows that depressive symptoms appear in as many as 23% of patients with MPNs [6].

One of the drugs recommended for the treatment of MDD is venlafaxine (VEN) [7]. This drug belongs to the group of serotonin (5-HT) and noradrenaline (NA) reuptake inhibitors (SNRIs). At low doses, it causes an increase in serotoninergic transmission. At higher doses (higher than 225 mg), it causes an increase in noradrenergic transmission [8,9]. VEN is now considered one of the most effective antidepressants, which is confirmed by studies describing its efficacy in treating resistant depression [10].

**Figure 1:**
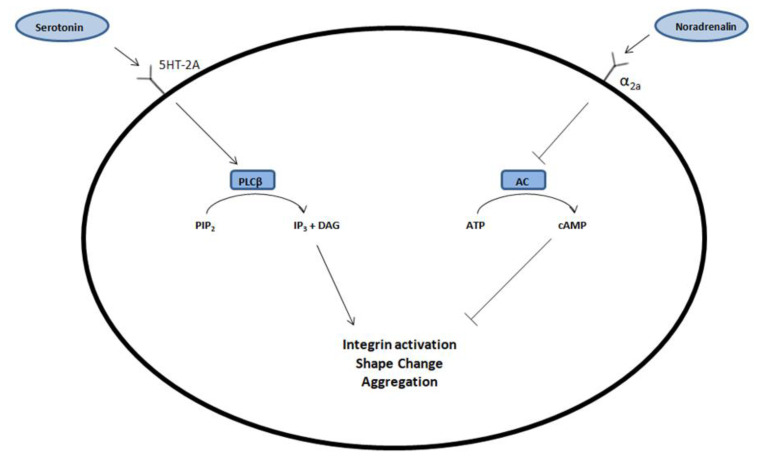
By binding to the 5HT-2A receptor on the surface of the blood platelets, serotonin (5-HT) causes an increase in the concentration of phospholipase Cβ (PLCβ). It causes conversion of phosphatidylinositol-4,5-bisphosphate (PIP2) to inositol-1,4,5-trisphosphate (IP-3), which activates integrin, changes shape of platelets, and at the end, causes an aggregation process. Noradrenalin (NA) exerts the same effect but by activating the α2a receptor, which inhibits adenylate cyclase (AC) and lowers the cAMP concentration. This process inhibits platelet aggregation. DAG—diacylglycerol.

Thus far, there are no scientific reports describing the effects of depression and antidepressant treatment on the production of the morphotic elements of blood in bone marrow; however, for many years, researchers’ attention has been drawn to the relationship between depression and changes in the number of platelets and mean platelet volume (MPV), as well as changes in the prothrombotic phenotype of platelets [11,12,13]. The potential cause of such changes is increased sympathetic activity in the course of depression [14]. This is due to the increased sensitivity of the nervous system to stressors in people with MDD. In this situation, stress factors, affecting the nervous system, increase corticotropin-releasing hormone (CRH) production in the pituitary gland. CRH then affects another part of our nervous system, the locus coeruleus, in which the production of NA increases in response to CRH stimulation. The NA produced there increases the sympathetic system’s activity and reduces parasympathetic activity [15,16]. Additionally, attention should be paid to the fact that two important biogenic amines, NA and 5-HT, have their own receptors on the platelet surface and are involved in the platelet aggregation process (Figure 1) [17].

This allowed the researchers to hypothesize that the use of drugs modulating the concentrations of 5-HT and NA may affect not only the number of platelets, but also their ability to aggregate [18,19]. One such drug is VEN.

Scientists have repeatedly pointed out the effect of drugs used in treating mental disorders on the number and activity of platelets. Such drugs include antipsychotics such as olanzapine or clozapine, which, apart from affecting the process of platelet aggregation, also affect their number, which can cause thrombocytopenia [20,21,22]. Other drugs that can significantly affect the platelet count are mood stabilizers, such as valproic acid [23] or lithium salts [24].

In our work, we present the case of VEN being added to therapy for a patient suffering from ET and MDD and consider the rarely reported effect of VEN lowering the platelet count.

## 2. Materials and Methods

In this publication, we describe the case of a 71-year-old patient suffering from MDD coexisting with ET. The retrospective case report uses data from the inpatient treatment conducted by three of the authors. The patient’s data were anonymized for this report. The patient was informed about the mechanism of action of VEN and its potential adverse effects; fully informed consent from the patient was obtained. We also prepare a narrative review of literature about the effect of VEN on platelet count and activity. To prepare this review, databases were reviewed to isolate reports according to the following key phrases: “venlafaxine”, “SNRI”, “platelets”, “platelet count”, and “essential thrombocythemia”. Publications published in 1997–2021 were taken into account.

## 3. Case Study

A 71-year-old patient was brought urgently by her family to the Clinical Department of Psychiatry in Tarnowskie Góry of the Medical University of Silesia. During the psychiatric examination in the emergency department, she had clearly pronounced anxiety and fear; she expressed reference, nihilistic, and sometimes catastrophic delusions; and she gave the impression that she was having auditory hallucinations. On admission, psychomotor retardation and depression of mood, of significant intensity, were also noticed. She required emergency pharmacotherapy and received 5 mg of diazepam and 2 mg of haloperidol. Later in the study, she was a bit calmer, but still suffered from severe psychomotor retardation and significant depression of mood. She was reluctant to make verbal contact—the statements were sparse, she answered the questions asked with single, short sentences, and psychotic symptoms still persisted. Because of these symptoms, she was admitted to the department with the diagnosis of a severe episode of depression with psychotic symptoms in the course of recurrent depressive disorder, according to The International Classification of Diseases (ICD-10).

The family reports showed that in the few months before hospitalization, her mental state was gradually deteriorating. Symptoms such as decreasing mood and psychomotor drive, deterioration of purposeful activity, sleep disorders, difficulty in focusing, and deterioration in cognitive functions, particularly memory disorders, appeared. Three days before admission, the family observed a significant deterioration of the mental state. Additional symptoms began to appear, such as mutism, akinesia, and limited responses to stimuli. According to the family, the intensity of verbal production with delusional content increased. Both the family and the patient on admission denied episodes of mania and hypomania in the past.

The analysis of the disease course indicated many years of psychiatric history. The patient was hospitalized twice in the past due to recurrent depressive disorder 17 and 18 years before the present hospitalization. Since her last hospital discharge, she had not experienced any exacerbations of disease symptoms requiring hospital treatment, but she was under constant outpatient psychiatric care.

Psychopharmacological therapy prescribed before admission consisted of 45 mg of mirtazapine per day, 20 mg of citalopram per day, and 7.5 mg of zopiclone as needed. For 6 years, she had been under the control of a hematology clinic due to ET. She confirmed the presence of the Jak V617F 12.5–31% mutation. Because of this, she was taking 1 mg of anagrelide per day in two divided doses. The last modification of hematological treatment was made 4 months before hospitalization.

The patient was also treated for hypertension for many years before hospitalization, and without any changes in the period before hospitalization, she took indapamide at a dose of 1.5 mg per day and 50 mg of metoprolol per day.

On admission to the hospital, the current treatment was modified. In the beginning, constantly admitted by the patient before hospitalization, citalopram was reduced and then, within 5 days, was discontinued due to its ineffectiveness in out-of-hospital treatment. Due to psychotic symptoms, haloperidol at a daily dose of 8 mg was added to the treatment, but because of persistent psychotic experiences, it was discontinued on the seventh day of hospitalization. Instead, olanzapine was included in the treatment at an initial dose of 5 mg per day to reach a dose of 10 mg after 10 days.

Because of the reduction in the intensity of psychotic experiences and persistent depressive symptoms on the 29th day of hospitalization, VEN was added to the current treatment at the initial dose of 37.5 mg. Due to the good clinical response, the dose was increased to 75 mg per day after 5 days. After 10 days, the dose of VEN was increased to 112.5 mg per day, but due to increased anxiety, the dose was reduced to 75 mg after 3 days. After 20 days, the dose was increased again to 112.5 mg per day, and after another 6 days, to 150 mg. After 92 days of VEN treatment, the dose was changed to 225 mg, which was maintained until the end of the observation period.

The diagnosis and the medical history were objectified after achieving a relative improvement in the mental state. The patient then admitted to the presence of episodes of hypomania in the past, which, due to the fulfillment of the diagnostic criteria according to ICD-10, resulted in the diagnosis being changed to bipolar disorder.

On the 76th day of hospitalization, aripiprazole was added to the treatment at an initial dose of 2.5 mg; after 7 days, the dose was increased to 5 mg per day.

During hospitalization, the hypertensive treatment was slightly modified due to persistently high blood pressure values. On day 22, 5 mg of perindopril was added to the treatment, and this dosage was increased to 10 mg per day on day 40.

The dosage of anagrelide was not modified throughout the observation period in accordance with the recommendations of the hematologist with whom the patient consulted several times during hospitalization. However, due to his recommendations, the patient’s platelet count was regularly monitored by performing a complete blood count.

Since VEN was added to the therapy, a gradual reduction in the number of platelets was observed. The intensity of this reduction was also dependent on the dose of VEN.

**Figure 2:**
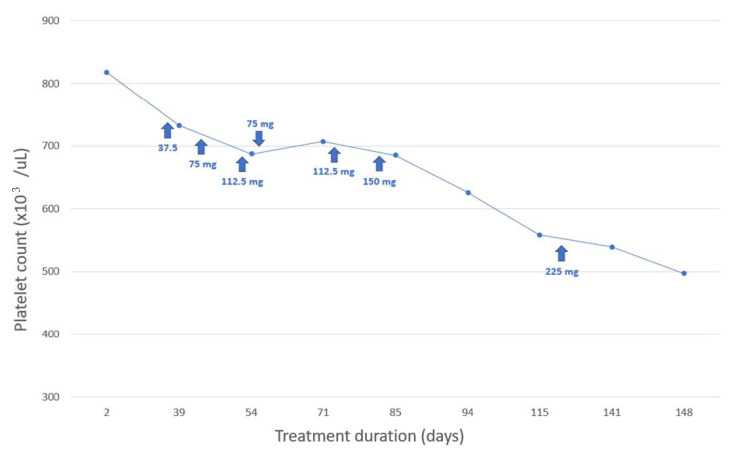
Relationship between platelet count and venlafaxine (VEN) treatment duration and dosage.

Detailed changes in platelet count over time and their dependence on VEN dosing are shown in Figure 2.

On the 139th day of hospitalization, lithium salts were included in the pharmacotherapy because of the diagnosis of bipolar disorder, significant improvement in mood, and reduction in depressive symptoms.

The case report focuses on the treatment period from the initiation of hospitalization to the 139th day of observation due to reports in the literature that lithium salts can significantly affect the platelet count [24] and interfere with previous observations.

## 4. Discussion

Thrombocytopenia is an adverse effect that we can observe after administering many drug groups. We can also find many examples of antidepressants that may inhibit the formation of blood platelets or intensify their destruction in the reticuloendothelial system of the liver and the spleen, which reduce their number in the blood [25,26,27].

However, one of the most frequently described mechanisms of platelet destruction is the mechanism that supports the increased production of anti-platelet antibodies, which is stimulated by the drugs taken [28]. One of the clinical studies that proved such an effect of antidepressant drugs is a study prepared by Song HR. et al., in which a reduction in platelet count was observed in patients receiving escitalopram or VEN, and such dependence was not observed in patients using bupropion (which belongs to the group of inhibitors of the dopamine and adrenergic receptor) [29]. It is also noteworthy that in this study, there was a stronger correlation between the use of the drug and a decrease in platelet count in the case of escitalopram than in the case of VEN, which may suggest greater importance for the inhibition of serotonergic over noradrenergic transmission.

Similar conclusions were drawn from a meta-analysis carried out in 2013, which included a group of 681 patients from two double-blind, randomized, placebo-controlled trials in which the safety of using another SNRI representative, duloxetine, was tested. Conclusions resulting from this meta-analysis show that in the group of patients taking duloxetine compared to the placebo group, a decrease in the number of platelets was observed [30].

The reverse relationship is shown in the study prepared by Gronau W. et al., in which a statistically significant increase in the platelet number after using VEN was visible [31].

Due to the presence of α2-adrenergic and 5HT-2A receptors on the surface of the platelets, it may be assumed that the effects of biogenic amine concentrations, such as NA and 5-HT, will be influenced by the platelet aggregation process. These assumptions were confirmed by an in vitro study conducted in 2012, which showed a statistically significant increase in the adhesion of platelets to fibrinogen after using VEN. Nevertheless, in this study, this effect did not result in an extension of the activated partial thromboplastin time and prothrombin time [32].

Despite the effect of SNRI described in the cited studies, the extent, and activity of platelets, their influence on the risk of bleeding, and the risk of cardiovascular events caused by their overactivity are still very unclear. To the best of our knowledge, the first report on the impact of VEN on the platelet aggregation process dates back to 2006. In the case study prepared by Sarma A. et al. regarding a patient in whom the administration of VEN caused the appearance of ecchymoses, the authors confirmed disorders of platelet aggregation, which they associated with the patient’s intake of VEN [33]. Similar symptoms were later described by Shaligram D. et al. in a patient taking desvenlafaxine [34]. These case reports prompted the researchers to conduct studies on larger groups of patients that assessed this previously unknown effect of SNRIs. In a study conducted on a group of 4136 patients after coronary artery bypass grafting, there was no relationship between patients taking selective serotonin reuptake inhibitor (SSRI) or SNRI before the procedure and the risk of excessive bleeding during the postoperative period and during longer follow-up. The same study showed a reduction in the risk of cardiac death, which, according to the authors, was probably related to the reduction in platelet activity [35]. Similar conclusions were drawn from a study by Smith MM et al. in a group of 1453 patients undergoing cardiac surgery who were taking SSRI or SNRI. An increased risk of bleeding and postoperative mortality was also not observed in the study [36]. A study prepared by Chachal P. et al. also did not reveal an increased risk of bleeding in patients receiving SNRI after endoscopic retrograde cholangiopancreatography [37].

On the other hand, in a group of patients with a left ventricular assist device (LVAD) who used SSRI and SNRI, a statistically significantly more frequent occurrence of gastrointestinal bleeding was observed compared to patients not using these drugs [38,39]. However, even the authors of this article highlight the important role of antidepressant therapy using SSRI or SNRI in this group of patients [40,41] and are not in favor of stopping it.

The case report prepared by us has some limitations. The most important limitation is polypharmacotherapy taken by the patient, which could interfere with the decrease in the number of platelets observed by us. However, to limit the impact of other drugs, in this article, we only present the period when the only modification of the therapy was the intensification of VEN doses. The second limitation is the initial misdiagnosis of MDD with bipolar disorder, which influenced the initial therapeutic decisions. However, this resulted from the patient’s serious condition on admission and from the fact that both the patient and the family denied the occurrence of hypomania episodes in the past.

## 5. Conclusions

Considering the results of scientific studies and the changes in the platelet count observed in the described patient, attention should be paid to the possible adverse effect of reducing the number of platelets in the group of patients taking VEN. Due to the probable mechanisms of this phenomenon, it is an adverse effect seemingly difficult to prevent in the case of chronic therapy. Therefore, ordering frequent complete blood count lab tests in such patients should be justified to detect it as early as possible and consider the further course of treatment. However, this topic requires further research to unambiguously determine the effect of SNRI on platelet count and activity.

## Data Availability

Data supporting the reported results of the treatment of the patient can be found in the author’s (M.P.) medical record and can be obtained upon request from the corresponding author.
